# Risk of cancer and serious disease in Danish patients with urgent referral for serious non-specific symptoms and signs of cancer in Funen 2014–2021

**DOI:** 10.1038/s41416-024-02620-y

**Published:** 2024-02-26

**Authors:** Rasmus Birkholm Grønnemose, Per Syrak Hansen, Søren Worsøe Laursen, Oke Gerke, Jakob Kjellberg, Jesper Lykkegaard, Clara Thye-Rønn, Poul Flemming Høilund-Carlsen, Peter Thye-Rønn

**Affiliations:** 1 Diagnostic Centre, Svendborg Hospital, Odense University Hospital, Svendborg, Denmark; 2 The Danish Cancer Society, Copenhagen, Denmark; 3 Department of Nuclear Medicine, Odense University Hospital, Odense, Denmark; 4 Department of Clinical Research, Faculty of Health Sciences, University of Southern Denmark, Odense, Denmark; 5 VIVE, The Danish Centre for Social Science Research, Copenhagen, Denmark; 6 Research Unit of General Practice, University of Southern Denmark, Odense, Denmark

**Keywords:** Cancer epidemiology, Epidemiology, Cancer epidemiology, Laboratory techniques and procedures, Predictive markers

## Abstract

**Background:**

In 2011, as the first European country, Denmark introduced the non-organ-specific cancer patient pathway (CPP) for patients presenting with non-specific symptoms and signs of cancer (NSSC). The proportion of patients with cancer over time is unknown.

**Methods:**

A retrospective cohort study of all patients with a NSSC-CPP investigational course in the province of Funen to the Diagnostic Centre in Svendborg from 2014 to 2021 was performed to evaluate the proportion of patients with cancer and serious disease over time.

**Results:**

A total of 6698 patients were referred to the NSSC-CPP of which 20.2% had cancer. While the crude referral rate increased from 114 per 100,000 people in 2014 and stabilised to around 214 in 2017–2021, the cancer detection rate of the total yearly new cancers in Funen diagnosed through the NSSC-CPP in DC Svendborg increased from 3 to 6%.

**Conclusions:**

With now high and stable conversion and crude referral rates, the NSSC-CPP is one of the largest CPPs in Denmark as measured by the number of new cancer cases found. Similar urgent referral programmes in other countries might fill an unmet medical need for patients presenting with serious non-specific symptoms and signs of cancer in general practice.

## Background

The United Kingdom and Denmark have for many years lagged behind comparable countries in terms of cancer survival. Although improvements have been observed especially for Denmark, both countries still lag behind otherwise similar countries in cancer survival [1–3]. This may in part be attributed to later stage at the time of diagnosis [4–6]. Optimising the route to reach a timely diagnosis is considered crucial for survival, as delay in diagnosis or treatment of even a few weeks can lead to increased mortality for most cancer types [7–10].

To accelerate timely diagnosis of cancer, several European countries have implemented urgent referral systems through organ-specific Cancer Patient Pathways (OS-CPP), including the 2-week wait referral pathway in the UK in 2000 [11] and the OS-CPPs in Denmark in 2007, Norway in 2015, and Sweden in 2015 [12–14]. These pathways are based on the referral of patients with alarm symptoms that give rise to suspicion of a specific cancer (e.g., rectal bleeding suggesting colorectal cancer). In the UK the pathway has a risk referral threshold of 3% [15], which is not the case in the Nordic countries.

However, around half of cancer patients do not present with alarm symptoms prior to diagnosis [16] and would therefore not prompt an urgent referral through these pathways. Patients without alarm symptoms thus experience an increased time to cancer diagnosis, as seen both in the UK and Denmark [16, 17]. To offer more patients a coordinated and more efficient diagnostic work-up and to reduce the amount of time that each patient have to wait and worry, Denmark implemented a new CPP in 2011–2012 for patients presenting with serious non-specific symptoms and signs of cancer (NSSC-CPP) [18]. These symptoms and signs include unintended weight loss, fatigue, unexplained fever, malaise, diffuse pain, the GP’s gut feeling for the patient having cancer, and abnormal blood samples. Based on the Danish model, a NSSC-CPP has also been implemented in Sweden and Norway [19, 20] and is being trialled in England at 10 pilot sites since 2016, where the English NSSC-CPP is expected to reach full population coverage by 2024 [21–23].

Previous studies of the Danish NSSC-CPP have shown a cancer prevalence in patients with serious non-specific symptoms of 11–20% [24–30] and a prevalence of serious non-malignant disease of 22% [29]. However, these studies have almost exclusively focused on the early years of the Danish NSSC-CPP from 2011 to 2015, and more recent studies are therefore needed to evaluate how the patient population has evolved over time, including the prevalence of malignant and serious non-malignant disease.

The current study involves a retrospective cohort referred to the Diagnostic Centre in Svendborg through the NSSC-CPP from 2014 to 2021 (both years included). The aim was to characterise the patient population of the NSSC-CPP over time in relation to disease prevalence and overall detection rate of new cancer cases in the province of Funen, covering 9% of the Danish population.

## Methods

### Study cohort

In Denmark, the general practitioner (GP) is the gatekeeper for referral to the secondary health sector, including referral to CPPs. When patients above 18 years old present at their GP with non-specific symptoms, and the GP suspects cancer, the GP can refer the patient to the NSSC-CPP. The NSSC-CPP in the Region of Southern Denmark is currently divided between 4 diagnostic centres (DC), where the DC in Svendborg receives referrals for all of Funen, including surrounding islands with a total catchment area population of ~499,000 residents (2021) [31] of which ~405,000 residents are above 18 years old. DC Svendborg opened in 2013 and up until May 2017 shared the referrals from Funen with Odense University Hospital, with patients from GPs in the municipalities of Odense and Northern Funen primarily being referred to DC Odense. Throughout the whole period, the municipally of Middelfart has been shared between DC Svendborg and DC Vejle.

Patients were referred to the NSSC-CPP by either the GP (incl. other private clinical specialists) or hospital departments. Prior to referral, the GP was required to perform a complete physical examination and obtain a specified blood sample panel as described in the NSSC-CPP [18], and the patient should not be a candidate for an organ-specific CPP. All NSSC-CPP patients with an investigational course in DC Svendborg that were referred from January 1, 2014 to December 31, 2021 were included in the study.

### Procedures performed at the diagnostic centre

At the DC, the patients are first seen by a nurse, who measures the patients’ weight and height, and asks about smoking habits (package years) and alcohol intake (units a week). A physician (either a GP in training or a consultant) then performs a physical examination and identifies any comorbidities or previous cancers from the medical records. The patients are usually referred to the radiology department for a dual-phase CT scan of the thorax-abdomen-pelvis (CT-TAP) on the same day, while patients considered at high risk or have specific symptoms are referred to the Department of Nuclear Medicine for a whole-body ^18^F-FDG PET/CT low dose scan on the following weekday. All results from CT-TAP scans are reviewed daily at a conference with a radiologist.

On the weekday after the scan, the patients are given a face-to-face or virtual consultation, where the results of the scan are presented together with a plan forward.

### Data collection

Data was collected manually from the electronic hospital records with a follow-up of 6 months from referral to include all investigational work-up related to the NSSC-CPP course, in which each patient was assigned a final diagnosis of either cancer, serious non-malignant disease, or non-serious disease. ICD-10 codes C00–96 (excluding non-melanoma skin cancers, C44), D469 (myelodysplastic syndrome), and D459 (polycythaemia vera) were categorised as cancers. In case of more than one primary cancer within 6 months of follow-up, the main cancer diagnosis was selected based on the severity of the cancer and associated treatment and/or course of disease (including cause of death). Only the main cancer diagnosis was used for further analyses. Serious disease was defined as a condition that requires further diagnostic follow-up and/or treatment in the secondary sector.

Date and source of referral to the DC (e.g., general practice, emergency department, other hospital department) were collected from the referrals. Information on sex and age was obtained from the civil registration number. From the electronic medical records, comorbidities were assessed by manual review, including previous cancers and current/former depression. Image diagnostics were assessed from the imaging description of the departments of radiology and nuclear medicine. An abnormal imaging finding was defined as a finding that needed treatment or further diagnostic work-up or could explain the symptoms of the patient. Results from blood samples were collected from the BCC software system (CGI, Canada). We chose to limit blood data collection in our database to 15 different blood test items comprising alanine transaminase (ALAT), alkaline phosphatase (ALP), B12 vitamin, bilirubin, calcium total, calcium ionised, C-reactive protein (CRP), serum creatinine, estimated glomerular filtration rate (eGFR), erythrocyte sedimentation rate (ESR), gamma-glutamyltransferase (GGT), haemoglobin, haemoglobin A1c (HbA1c), lactate dehydrogenase (LDH) and M component.

### Statistics and data reporting

Descriptive statistics were applied according to data type. Continuous and normally distributed variables were summarised by mean and standard deviation (SD), whereas continuous variables otherwise were reported by median and interquartile range (IQR). Frequencies and respective percentages described categorical variables. Exploratory testing for differences between cancer and non-cancer patient characteristics was done for continuous variables with Student’s unpaired *t* test and for categorical variables with Z-test for two population proportions and Chi-squared test. Results from blood tests from patients who had been categorised as cancer and non-cancer cases were compared with uni- and multivariate logistic regression for continuous variables, while categorical variables were compared with odds ratios with 95% confidence intervals. Level of statistical significance was 5% (two-sided) without adjustment for multiple testing. All analyses and figures were compiled in R software (version 4.2.2, R Core Team 2023) using the libraries ggplot2, dplyr, tidyr, sunburstR and ggthemes.

The reporting of the study was guided by the Strengthening the Reporting of Observational Studies in Epidemiology (STROBE) statement [32].

## Results

### Study population

A total of 6698 investigational courses were completed during the study period from 2014 to 2021. Of these, 6456 represented unique patients, while the remaining 242 courses represented patients with at least one previous NSSC-CPP course at least 1 month apart with a median of 608 days between courses (IQR: 330–1079). These investigational courses were regarded as separate entities in the following analyses. The yearly number of referred patients increased from 299 in 2014 and stabilised to around 1000–1100 in 2017–2021, which equals a crude referral rate of 114 and around 214 per 100,000 people, respectively, when adjusting for the difference in catchment area population for DC Svendborg between 2014 and 2021 (Table 1).

**Table 1:** Referrals, age, Charlson Comorbidity Index and diagnosis listed per year.

	2014	2015	2016	2017	2018	2019	2020	2021
Referral								
Number of referrals	299	547	688	885	1028	1038	1119	1094
Catchment area population	261,846	262,068	263,204	494,049^a^	496,243	498,481	498,506	499,419
Crude referrals per 100,000	114	209	261	212^b^	207	208	224	219
Age								
Median (IQR)	70 (61–78)	70 (60–78)	68 (60–75)	68 (57–76)	70 (59–77)	70 (58–77)	69 (58–77)	71 (61–77)
Charlson Comorbidity Index								
Median CCI score (IQR)	1 (1–3)	2 (1–3)	2 (1–3)	2 (1–3)	2 (0–3)	2 (1–3)	1 (0–2)	2 (1–3)
Mean CCI score (SD)	2.1 ± 1.8	2.3 ± 1.8	2.1 ± 1.7	2.0 ± 1.7	1.9 ± 1.7	2.0 ± 1.9	1.7 ± 1.7	2.0 ± 1.8
Diagnosis								
Malignant disease, % (*n*)	39.5 (118)	25.2 (138)	22.8 (157)	20.1 (178)	18.5 (190)	18.5 (192)	15.4 (172)	18.9 (207)
Serious non-malignant disease, % (*n*)	26.4 (79)	30.3 (166)	36.0 (248)	25.5 (226)	25.6 (263)	25.0 (259)	15.1 (169)	20.7 (226)

Data on catchment area population are provided by Statistics Denmark [31] as assessed on 1st January each year and include all citizens regardless of age with residence in each municipality.

^a^The catchment area population of the Diagnostic Centre in Svendborg increased in May 2017, when the municipalities of Odense and Northern Funen were included in the catchment area. Only the population of the new catchment area is shown for 2017.

^b^The crude referral for 2017 was calculated using the mean catchment area population for the year thus adjusting for the smaller catchment area population of January until April 2017 (catchment area population: 264,040).

### Patient characteristics and comorbidities

The median age of the patients was 70 years (IQR: 59–77) with 49.4% being male (Table 2) with patients subsequently being diagnosed with cancer having a significantly higher age than non-cancer patients. The gender distribution was close to equal across all age groups, while the general age structure was skewed towards the patients of 60 years and above as compared to the general population of Funen (Supplemental Fig. 1).

**Table 2:** Characteristics of all NSSC-patients and these divided into those diagnosed with or without cancer within 6 months from referral to NSSC-CPP at the Diagnostic Centre in Svendborg, Denmark.

	All	Cancer	No cancer	*P* value
% (*N*)	100 (6698)	20.2 (1352)	79.8 (5346)	–
Referred by				
General practice	80.4 (5383)	75.5 (1021)	81.6 (4362)	<0.001
Emergency department	3.6 (243)	5.2 (70)	3.2 (173)	
Other hospital department	12.0 (803)	11.2 (152)	12.2 (651)	
Unknown	4.0 (269)	8.1 (109)	3.0 (160)	
Gender				
Male	49.4 (3312)	53.5 (723)	48.4 (2589)	<0.001
Age				
median (IQR)	70 (59–77)	73 (66–79)	68 (57–76)	<0.001
Groups				
18–39	4.1 (274)	0.7 (9)	5.0 (265)	<0.001
40–54	13.8 (921)	5.6 (76)	15.8 (845)	
55–69	31.9 (2137)	28.1 (380)	32.9 (1757)	
70–79	32.6 (2184)	40.9 (553)	30.5 (1631)	
80+	17.6 (1182)	24.7 (334)	15.9 (848)	
Body mass index (BMI)				
Median (IQR)	24.7 (21.9–28.2)	24.7 (22.3–27.9)	24.7 (21.7–28.3)	0.374
Smoking status				
Never	39.4 (2170)	35.5 (395)	40.3 (1775)	0.003
Package years median (IQR)^a^	30 (15–40)	30 (15–45)	26 (14–40)	<0.001
Alcohol consumption per week				
No alcohol intake	52.3 (2956)	50.6 (571)	52.7 (2385)	0.208
Units per week, median (IQR)^b^	7 (5–14)	7 (5–14)	7 (5–14)	0.071
Above national guidelines^c^	17.6 (994)	17.2 (194)	17.7 (800)	0.697
Chronic diseases				
Hypertension	47.3 (3044)	54.3 (675)	45.7 (2369)	<0.001
Myocardial infarct	5.4 (347)	5.3 (66)	5.4 (281)	0.889
Congestive heart failure	8.7 (557)	9.3 (116)	8.5 (441)	0.368
Peripheral vascular disease	41.1 (2640)	49.3 (613)	39.1 (2027)	<0.001
Cerebrovascular disease	12.4 (799)	13.5 (168)	12.2 (631)	0.211
Dementia	2.0 (128)	2.7 (33)	1.8 (95)	0.040
Chronic pulmonary disease	21.1 (1358)	18.3 (228)	21.8 (1130)	0.007
Connective tissue disease	12.0 (773)	7.8 (97)	13.0 (676)	<0.001
Ulcer disease	29.6 (1903)	29.3 (364)	29.7 (1539)	0.779
Mild liver disease	5.5 (355)	5.4 (67)	5.6 (288)	0.779
Diabetes without complications	11.4 (731)	11.3 (141)	11.4 (590)	0.920
Diabetes without/end-organ damage	3.7 (241)	4.2 (52)	3.6 (189)	0.317
Hemiplegia/paraplegia	0.8 (54)	0.6 (7)	0.9 (47)	0.298
Moderate/severe renal disease	14.2 (914)	19.5 (242)	13.0 (672)	<0.001
Moderate/severe liver disease	3.3 (215)	4.3 (53)	3.1 (162)	0.034
HIV/AIDS	0.0 (0)	0.0 (0)	0.0 (0)	–
Charlson Comorbidity Index^d^				
Median CCI score (IQR)	2 (1–3)	2 (1–3)	2 (1–3)	–
Mean CCI score ± SD	1.96 ± 1.78	2.14 ± 1.78	1.92 ± 1.78	<0.001
0	22.7 (1458)	18.2 (226)	23.8 (1232)	<0.001
1	25.1 (1610)	24.5 (304)	25.3 (1306)	
2	20.8 (1336)	22.4 (278)	20.5 (1058)	
≥3	31.3 (2010)	34.9 (434)	30.5 (1576)	
Other				
Former or current depression	14.7 (921)	13.5 (166)	15.0 (755)	0.184
Previous cancer^e^	17.1 (1087)	22.4 (281)	15.8 (806)	<0.001
Weight loss	64.1 (3976)	61.0 (738)	64.9 (3238)	0.011

Data are generally presented as percentages (n) or when indicated as median with interquartile range (IQR) or means ± standard deviation (SD). On average, the variables had 5.3% missing values with the highest percentage of missing values being smoking (17.7%), alcohol consumption (15.5%), and body mass index (13.4%).

^a^Median package years were calculated only for former or current smokers.

^b^Median alcohol intake was calculated only for patients with weekly intake of alcohol. One unit of alcohol equals 12 g/15 ml of pure alcohol.

^c^National guidelines for alcohol consumption are currently above 10 units of alcohol per week for both men and women.

^d^The Charlson Comorbidity Index (CCI) score was calculated as specified in Charlson et al. [33] without scoring for cancer. Hypertension is not included in the original CCI.

^e^Previous cancers include all cancers except non-melanoma skin cancers. Patients with multiple previous cancers or recurrences were only counted as one.

Of all patients, 39.4% had never smoked (Table 2), while current or former smokers had a median number of package years of 30 (IQR: 15–40). Patients with cancer had a significant higher proportion of current or former smokers, and these had a significantly higher number of package years than non-cancer patients. More than half of the total population did not have a daily intake of alcohol (52.3%), while 17.6% had an intake above the Danish guidelines (10 units per week for both genders). No significant differences were seen between cancer and non-cancer patients in regard to alcohol consumption. Of the 6698 patients, 22.7% had no comorbidities as defined by the Charlson Comorbidity Index (CCI) [33], while 52.1% had a CCI score of ≥2 with cancer patients having significantly more comorbidities than non-cancer patients, though these differences have not been adjusted for possible confounders such as higher age or smoking. The major comorbidities were hypertension (47.3%), peripheral vascular disease (41.1%), ulcer disease (29.6%), chronic pulmonary disease (21.1%), and moderate to severe renal disease (14.2%). Of note, cancer patients had a significantly higher proportion having hypertension, peripheral vascular disease, dementia, renal disease, and moderate to severe liver disease while also having a significantly lower proportion of connective tissue disease and chronic pulmonary disease than non-cancer patients. Depression is known to be associated with an increased risk of cancer and diagnostic delay [34–36]. However, in our study, where 14.7% of the overall population had current or former depression, this was not associated with an increased risk of cancer. On the other hand, 17.1% of all patients had previously been diagnosed with cancer (excluding non-melanoma skin cancer) with a significantly higher proportion in cancer patients. The median measured body mass index at first visit was 24.7 (IQR 21.9–28.2) with no difference between groups, while 64.1% reported a weight loss, which was interestingly found less frequently in cancer patients. The overall referred patient population did not change from 2014 to 2021 in regard to age or CCI, both of which remained almost constant throughout the whole period (Table 1).

### Investigational procedures and findings

The median duration of the investigational course at DC Svendborg was 7 days (IQR: 5–14) from referral with 91% of the investigational courses being finished within the Danish NSSC-CPP 22-day guidance. Diagnostic imaging was performed within 12 weeks prior to first visit or during the investigational course in 94.7% of the patients with the preferred imaging type being CT (82.0%), X-ray (22.9%), PET/CT (16.4%), and ultrasound (6.9%). A total of 6.5% of the patients were examined with both a CT and a PET/CT scan. Of patients with diagnostic imaging within 12 weeks prior to first visit or during the investigational course, 58% with CT, 18% with X-ray, 77% with PET/CT, and 42% with ultrasound had abnormal findings with a total of 60% of the patients having at least 1 abnormal finding. Of the remaining 40% of patients who did not have abnormal imaging, 3.9% had cancer with the majority being haematological cancers (51%) or localised prostate cancer (16%). Bone marrow biopsy was performed in 4% of the patients.

When looking at the nominal levels of the included blood test items using logistic regression (Table 3), higher levels of ALAT, B12, ALP, bilirubin, LDH, CRP, GGT, creatinine and ESR were associated with increased odds of having cancer. On the other hand, higher haemoglobin and eGFR were associated with significantly lower odds ratio of having cancer. No significant difference between the groups was seen for total and ionised calcium or for HbA1c, and significant results for ALAT, creatinine, and eGFR found in the univariate logistic regression were no longer significant after adjusting for possible confounders for cancer (age, gender, smoking and alcohol consumption).

**Table 3:** Blood test items as predictors of cancer.

Blood test item	% (*N*)	Units	Median (IQR)			Univariate analysis			Adjusted analysis^a^		
			Total	Cancer	No cancer	OR	95% CI	*P* value	Adjusted OR	95% CI	*P* value
ALAT	94.4 (6320)	U/l	21 (16–31)	20 (15–31)	22 (16–32)	1.001	1.000, 1.001	**0.027**	1.001	1.000, 1.001	0.108
ALP	94.1 (6305)	U/l	79 (64–105)	93 (71–144)	77 (62–100)	1.004	1.003, 1.004	**<0.001**	1.003	1.003, 1.004	**<0.001**
B12	69.7 (4667)	pmol/l	352 (262–490)	389 (277–578)	345 (260–477)	1.001	1.001, 1.001	**<0.001**	1.001	1.001, 1.001	**<0.001**
Bilirubin	91.0 (6093)	µmol/l	7 (5–11)	7 (5–11)	7 (5–11)	1.004	1.002, 1.006	**0.001**	1.003	1.001, 1.006	**0.006**
Ca total	51.1 (3421)	mmol/l	2.37 (2.30–2.44)	2.37 (2.29–2.45)	2.37 (2.30–2.44)	1.365	0.768, 2.433	0.290	1.465	0.802, 2.682	0.214
Ca^++^	58.3 (3903)	mmol/l	1.27 (1.23–1.31)	1.27 (1.23–1.31)	1.27 (1.23–1.30)	1.937	0.960, 3.793	0.058	1.878	0.883, 3.878	0.092
Creatinine	95.3 (6381)	µmol/l	75 (63–90)	77 (64–96)	74 (63–89)	1.003	1.001, 1.004	**0.004**	0.999	0.997, 1.001	0.324
CRP	92.5 (6193)	mg/l	4 (1–19)	16 (3–48)	3 (1–12)	1.011	1.010, 1.013	**<0.001**	1.010	1.008, 1.011	**<0.001**
eGFR	95.3 (6381)	ml/min/1.73 m^2^	88 (69–98)	83 (63–94)	89 (71–99)	0.989	0.986, 0.992	**<0.001**	1.000	0.997, 1.004	0.894
ESR	42.6 (2855)	mm	16 (7–42)	33 (12–66)	14 (6–37)	1.015	1.012, 1.018	**<0.001**	1.013	1.009, 1.016	**<0.001**
GGT	90.5 (6064)	U/l	34 (20–74)	40 (22–111)	32 (19–68)	1.001	1.000, 1.001	**<0.001**	1.000	1.000, 1.001	**0.002**
Hb	94.9 (6356)	mmol/l	8.3 (7.4–8.9)	7.8 (6.8–8.6)	8.3 (7.6–9.0)	0.715	0.681, 0.751	**<0.001**	0.778	0.736, 0.822	**<0.001**
HbA1c	63.7 (4265)	mmol/mol	38 (34–42)	39 (35–44)	37 (33–42)	1.001	0.997, 1.005	0.597	0.999	0.993, 1.005	0.765
LDH	85.4 (5721)	U/l	199 (173–231)	215 (181–275)	195 (171–224)	1.004	1.003, 1.004	**<0.001**	1.004	1.003, 1.005	**<0.001**

*ALAT* alanine transaminase, *ALP* alkaline phosphatase, *CI* confidence interval, *CRP* C-reactive protein, *eGFR* estimated glomerular filtration rate, *ESR* erythrocyte sedimentation rate, *GGT* gamma-glutamyltransferase, *Hb* haemoglobin, *HbA1c* haemoglobin A1c, *LDH* lactatdehydrogenase, *OR* odds ratio.

Reference intervals can be found in Supplemental Table 1. Percentage (*N*) shows the percentage and number of non-missing values for each variable. For each blood test item, the median values with interquartile ranges (IQR) are shown for both the total of all patients, cancer patients only and non-cancer patients.

Significant *P* values are highlighted in bold.

^a^Odds ratio after adjusting for age, gender, smoking (package years), and alcohol consumption per week using multivariate logistic regression analysis.

The nominal values were then dichotomised into either abnormal blood values or critical blood values as defined as blood values outside reference intervals or values raising high suspicion of serious disease including cancer, respectively (see Supplemental Table 1 for more details). Here at least one or at least five abnormal blood values were seen in 81.2% and 19.1% of the total number of patients (Table 4), respectively, and these were associated with increased odds of having cancer (odds ratios (OR) of 3.2 and 2.7, respectively). The five most frequent abnormal blood findings were elevated ESR (38.5%), lowered haemoglobin (33.4%), elevated CRP (33.2%), elevated LDH (28.2%) and elevated ALP (24.9%). Of the abnormal findings, values such as low haemoglobin, high B12, low eGFR and high total and ionised calcium were associated with increased odds of having cancer (Table 4). Conversely, low B12, high HbA1c and low total and ionised calcium were not correlated with cancer.

**Table 4:** Abnormal or critical blood values (as defined in Supplemental Table 1) in all patients and in patients with or without cancer.

Blood test item	Abnormal values						Critical values					
	Total	Cancer	No cancer	OR	95% CI	*P* value	Total	Cancer	No cancer	OR	95% CI	*P* value
No abnormal values^a^	18.8 (1139)	8.0 (94)	21.4 (1045)	0.317	0.254, 0.396	**<0.001**	54.6 (3301)	31.5 (371)	60.1 (2930)	0.304	0.266, 0.349	**<0.001**
At least 1 abnormal^a^	81.2 (4912)	92.0 (1085)	78.6 (3827)	3.152	2.525, 3.934	**<0.001**	45.4 (2750)	68.5 (808)	39.9 (1942)	3.286	2.869, 3.763	**<0.001**
At least 5 abnormal^a^	19.1 (1155)	33.6 (396)	15.6 (759)	2.741	2.374, 3.164	**<0.001**	2.1 (126)	5.8 (68)	1.2 (58)	5.080	3.557, 7.255	**<0.001**
ALAT	9.8 (617)	12.4 (152)	9.1 (465)	1.417	1.166, 1.722	**<0.001**	4.0 (254)	6.4 (78)	3.5 (176)	1.909	1.451, 2.511	**<0.001**
ALP	24.9 (1573)	39.9 (486)	21.4 (1087)	2.443	2.139, 2.790	**<0.001**	6.2 (394)	15.3 (186)	4.1 (208)	4.228	3.431, 5.210	**<0.001**
B12 low	9.5 (443)	9.1 (79)	9.6 (364)	0.946	0.733, 1.222	0.672	2.3 (109)	2.1 (18)	2.4 (91)	0.864	0.518, 1.441	0.576
B12 high	15.5 (722)	23.6 (205)	13.6 (517)	1.966	1.639, 2.359	**<0.001**	4.1 (190)	8.9 (77)	3.0 (113)	3.180	2.357, 4.291	**<0.001**
Bilirubin	4.3 (265)	6.5 (77)	3.8 (188)	1.741	1.325, 2.288	**<0.001**	1.8 (112)	4.2 (50)	1.3 (62)	3.436	2.354, 5.015	**<0.001**
Ca total Low	6.0 (205)	6.9 (41)	5.8 (164)	1.207	0.847, 1.720	0.299	0.5 (16)	0.5 (3)	0.5 (13)	1.101	0.313, 3.876	0.881
Ca total High	6.2 (211)	8.4 (50)	5.7 (161)	1.525	1.096, 2.123	**0.012**	0.9 (30)	2.7 (16)	0.5 (14)	5.574	2.705, 11.483	**<0.001**
Ca^++^ low	5.4 (209)	5.0 (41)	5.5 (168)	0.904	0.637, 1.284	0.574	0.8 (33)	0.7 (6)	0.9 (27)	0.827	0.340, 2.009	0.674
Ca^++^ high	15.6 (610)	18.4 (152)	14.9 (458)	1.290	1.054, 1.578	**0.014**	3.6 (142)	5.6 (46)	3.1 (96)	1.831	1.277, 2.625	**0.001**
Creatinine	16.7 (1068)	21.1 (259)	15.7 (809)	1.435	1.228, 1.678	**<0.001**	–	–	–	–	–	–
CRP	33.2 (2053)	57.4 (689)	27.3 (1364)	3.587	3.149, 4.086	**<0.001**	24.0 (1484)	45.7 (548)	18.7 (936)	3.643	3.186, 4.165	**<0.001**
eGFR	16.3 (1037)	20.9 (257)	15.1 (780)	1.484	1.268, 1.736	**<0.001**	2.1 (134)	2.0 (24)	2.1 (110)	0.914	0.585, 1.428	0.914
ESR	38.5 (1098)	57.1 (317)	34.0 (781)	2.587	2.142, 3.125	**<0.001**	21.2 (605)	33.7 (187)	18.2 (418)	2.285	1.861, 2.807	**<0.001**
GGT	20.7 (1255)	27.8 (318)	19.0 (937)	1.634	1.410, 1.894	**<0.001**	11.3 (687)	17.1 (196)	10.0 (491)	1.863	1.556, 2.230	**<0.001**
Hb	33.4 (2121)	49.2 (603)	29.6 (1518)	2.303	2.028, 2.615	**<0.001**	6.3 (400)	12.5 (153)	4.8 (247)	2.819	2.280, 3.485	**<0.001**
HbA1c	12.3 (523)	12.2 (97)	12.3 (426)	0.995	0.786, 1.259	0.965	3.8 (164)	3.3 (26)	4.0 (138)	0.818	0.534, 1.252	0.355
LDH	28.2 (1611)	39.5 (446)	25.4 (1165)	1.924	1.679, 2.206	**<0.001**	11.1 (637)	24.5 (276)	7.9 (361)	3.798	3.194, 4.515	**<0.001**
M component	9.4 (395)	16.1 (133)	7.7 (262)	2.287	1.827, 2.862	**<0.001**	–	–	–	–	.	–

*ALAT* alanine transaminase, *ALP* alkaline phosphatase, *CRP* C-reactive protein, *eGFR* estimated glomerular filtration rate, *ESR* erythrocyte sedimentation rate, *GGT* gamma-glutamyltransferase, *Hb* haemoglobin, *HbA1c* haemoglobin A1c, *LDH* lactate dehydrogenase.

Data are presented as percentages (*n*) or as odds ratio (OR) with 95% confidence intervals.

Significant *P* values are highlighted in bold.

^a^Only patients with values for at least ten blood test items were included in the indicated subanalyses.

The most frequent critical values were highly elevated CRP (24.0%), ESR (21.2%), GGT (11.3%), and LDH (11.1%) and very low Hb (6.3%). Furthermore, critical values such as very low Hb (OR 2.8), very high CRP (OR 3.6), very high B12 vitamin (OR 3.2), and very high total (OR 5.6) and ionised calcium (OR 1.8) were associated with cancer, but values such as very low eGFR and very high HbA1c were not.

In a cross-tabulation analysis between blood and imaging findings (Supplemental Table 2), the highest association with cancer were seen in patients having both at least one abnormal imaging and at least one abnormal blood finding (33.1% cancer proportion), whereas the lowest association was seen in the group with no abnormal blood and imaging findings (1.5% cancer proportion).

### Investigational outcome

**Fig. 1:**
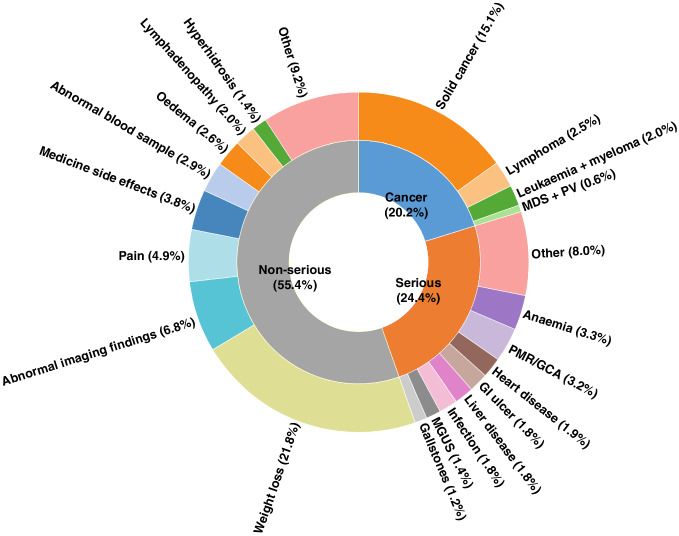
Sunburst plot of the diagnosis distribution of malignant disease, serious non-malignant disease, and non-serious disease/finding. For serious non-malignant disease, the group “Other” compromises a number of rare conditions with a less than 1% prevalence, including arthritis (0.7%), chronic obstructive pulmonary disease (0.7%), benign tumours (0.5%), and more. For non-serious disease/finding, the group “Other” includes a number of conditions with a less than 1% prevalence including goitre (0.9%), unexplained fever (0.6%), irritable bowel syndrome (0.6%), abdominal hernia (0.6%), and more. MDS/PV myelodysplastic syndrome/polycythaemia vera, PMR/GCA polymyalgia rheumatica/giant cell arteritis, GI ulcer gastrointestinal ulcer, MGUS monoclonal gammopathy of undetermined significance.

**Fig. 2:**
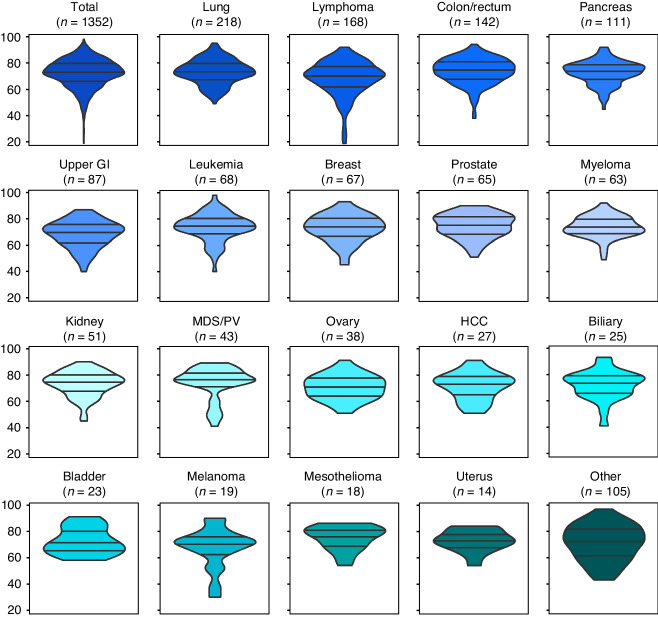
Violin plots displaying the age distribution of different cancer types diagnosed within 6 months from NSSC-referral to the Diagnostic Centre, Svendborg, from 2014 to 2021. The category “Other” embraces CUP or unknown (61 patients), head and neck cancers (15), sarcomas (12), thyroid cancers (6), brain cancers (6), vulva cancers (<5), testicular cancers (<5), and adrenal cancers (<5). CUP cancer of unknown primary, HCC hepatocellular carcinoma, MDS/PV myelodysplastic syndrome/polycythaemia vera, upper GI upper gastrointestinal tract which includes the oesophagus, gastroesophageal junction, stomach, and small intestine.

A total of 1352 patients (20.2%) were diagnosed with a malignant disease (Fig. 1) of which 8.6% (108 of 1253 patients with known previous cancer status) represented a recurrence from a previous cancer. Of the 1352 cancer patients, 22 patients had at least one additional primary cancer diagnosed within the 6 months of follow-up (see Supplemental Table 3 for details). The five most frequent types of main cancers found were lung cancer (3.3% of all referred patients), lymphoma (2.5%), colon/rectum cancer (2.1%), pancreas cancer (1.7%), and upper gastrointestinal cancer (1.3%) (Fig. 2). The five most common recurrent cancers were breast cancer (33.3% of all recurrent cancers), colorectal cancer (14.8%), prostate cancer (8.3%), malignant melanoma (7.4%), and upper GI cancers (5.6%). Of these five cancer types, recurrent cancers contributed to 58.1% (breast), 12.1% (colorectal), 14.5% (prostate), 42.1% (malignant melanoma), and 7.4% (upper GI) of the total cancers found of each type, respectively.

The overall age distribution of the cancer cases was skewed towards the elderly with cancers such as lung, prostate, and myeloma almost exclusively being diagnosed in patients above 60, while certain cancers such as lymphoma were also found in the youngest age groups (Fig. 2).

Of the remaining patients, 1636 patients (24.4% of all referred patients) were diagnosed with serious non-malignant disease, while 3710 patients (55.4%) were diagnosed with a non-serious disease or finding (Fig. 1). The major serious non-malignant diseases were anaemia (3.3% of all referred patients), PMR/GCA (3.2%), heart disease (1.9%), ulcer of the upper gastrointestinal tract (1.8%), and liver disease (1.8%). The most frequent non-serious disease/finding was unexplained weight loss (21.8%), abnormal imaging findings (6.8%), pain (4.9%), medical side effects (3.8%), and abnormal blood samples (2.9%).

The proportion of malignant and serious disease, however, changed year-over-year. The initial proportion of malignant disease fell from 39% in 2014 to 25% in 2015 and stabilised around 15–20% in 2017–2021. The proportion of serious disease on the other hand fluctuated from 26% in 2014 up to 36% in 2016 and down to 15–21% in 2020–2021 (Table 1), of which the latter period coincided with the COVID-19 epidemic and lockdown in Denmark.

## Discussion

In this study, we provide data of how the Danish urgent referral programme for patients with non-specific symptoms and signs of cancer has evolved from 2014 up until 2021 in a local setting in the area of Funen covering 9% of the Danish population. Here we have in recent years reached a high and stable number of referrals together with a high and stable conversion rate of around 20% of referred patients that turned out to have cancer, which is equivalent to around 6% of all new cancer cases on Funen. Furthermore, 24% of the patients were found to have serious non-malignant disease that required further diagnostic work-up and/or treatment. Our findings show that the NSSC-CPP has been effectively implemented as a clear proposition to the GPs and hospital departments as evident in a stable crude referral rate and conversion rate despite the highly heterogeneous symptomatic patient pool with multiple comorbidities and symptoms. Given the continuous medical need for fast and coordinated diagnostic intervention for this patient population, the NSSC-CPP is now an essential part of the Danish cancer programme serving a large dynamic patient population pool, and similar urgent referral pathways in other countries might improve the diagnostic work-up for similar patients in these countries. Thus, our study of an 8-year period can serve as a reference for other countries to what patient characteristics and clinical outcomes to expect when introducing a NSSC-CPP.

The most prominent strength of this study is the cohort size of 6698 investigational courses that includes all patients with an investigational course in the DC from 2014 to 2021, thus reflecting the clinical setting of the NSSC-CPP. In this way, the study is also the largest single-centre study in Denmark ever reported and largest Danish study of the NSSC-CPP that does not rely on automated gathering of diagnoses or patients from national registries. In our study, the use of manual revisions of journals enables the solving of discrepancies and to identify the most clinically relevant diagnosis related to the investigational course also in difficult patients with no clear malignant or serious disease or in case of underreported diagnoses or comorbidities in the patient index. However, the need for manual revision of each case also highlights the lack of outcome monitoring of the NSSC-CPP in Denmark, which currently only includes the number of investigational courses inside or outside the specified maximum recommended time interval of 22 days from first visit at the DC to end of the investigational course.

However, there are some limitations that are important to be highlighted. The study has a retrospective design and thus relies on electronic patient files with no way to correct missing or incorrect data, which could include underreported or misinterpreted comorbidities both in the patient index and in the record.

Another limitation is the use of the regional electronic health record system, which only includes records of the secondary public health system in the Region of Southern Denmark. This could lead to an underreporting of cancer cases or other serious diseases that might have been found in another region (for example due to the patient moving address) or from diagnosis in the private hospital sector. However, these concerns might only be minor, as for example the number of people moving away from the Region of Southern Denmark is limited to around 1.7% per year during the period [37]. Also, though the records from private hospitals are not available in the regional health record system, the private hospitals make up less than 2% of the overall Danish publicly funded hospital activities [38], and the pathology results are furthermore still visible in the regional pathology system.

Additional improvements and implications of this study could be the inclusion of more blood test items that might also be correlated with cancer or other serious diseases. These could include the full NSSC-CPP blood sample package as recommended by the Danish Health Authority. Together, the combined data would enable risk modelling by both multiple logistic regression models and artificial intelligence models, the latter of which was used in a recent Danish study [39]. Such a tool could thus serve to support the clinician in diagnostic decisions, including which imaging modality would be most appropriate or which patients to see in the NSSC-CPP. Importantly, most of the included blood test items in this study did indeed show an correlation with cancer as also seen in other studies [40], which underscores the possibilities with more advanced approaches. In addition, while detection of unexpected weight loss in general practice has been associated with cancer in the general population [41], we see a negative correlation in our selected population referred to the NSSC-CPP with various serious symptoms, GP’s gut feeling, and/or abnormal blood samples besides weight loss. In this way, our study also differs from previous studies of the NSSC-CPP in Denmark, Sweden, and the UK that were not able to find either a positive or a negative association between weight loss and cancer [19, 22, 24, 29, 42]. This highlights the need for inclusion of other symptoms that could help to identify specific symptoms or combinations of symptoms that might be more predictive of cancer than weight loss alone in patients with serious non-specific symptoms.

A number of NSSC-CPP studies has previously been conducted in Denmark [43]. However, these have almost exclusively been based on the early years of the urgent referral programme from 2011 up until the end of 2015 with timeframes usually of 1–3 years [24–30, 44, 45], the latter of which limits the possibilities of looking at differences over longer periods of time. Our study is the longest and largest single-centre study as measured by patients with 6698 in total, whereas previously single-centre studies have been in the range of ~300–1300 [24–26, 29]. Our study thus provides more reliable information about the frequencies of both common and rarer types of cancers and other serious diseases in this patient group and how the proportions of malignant and serious non-malignant disease have changed over time up until recently.

Consistent with our findings of 20.2% cancer cases, previous Danish studies have shown a cancer frequency in the range of 13–20% for most studies [24–26, 29, 30] with an outlier of 11% in a national registry study of nearly 24,000 patients that had almost no comorbidities and markedly different cancer types [28]. However, as seen in our study, these numbers might not be stationary, where we have seen an initial proportion of patients with cancer in 2014–2015 of 25–39%, which has been reduced to a stable 15–20% in recent years. Keeping in mind that our Diagnostic Centre opened in 2013, this substantial difference over time might be explained by teething problems in the early years where the referral threshold and diagnostic proposition of the NSSC-CPP was being worked out in close cooperation with general practice, hospital departments, outpatient clinics, and emergency departments. Thus, studies with more recent data might reflect the current conversion rate of the NSSC-CPP more accurately. Indeed, as seen in our study, as the crude referral rises and stabilises, we see a decline followed by a stabilisation in the proportion of patients with cancer. This means that the GPs and secondary care are using the NSSC-CPP more often, and thus less patients over time might be handled outside of an urgent referral programme, which can lead to faster cancer diagnosis and improved survival [46–48].

In the UK, pilot studies of the newly established Rapid Diagnostic Centres have shown a conversion rate of 8% [22] with individual sites showing conversion rates of 7–12% [42, 49, 50]. Though all are below their Danish counterparts, they all are above the NICE guideline of 3% [15]. Serious non-malignant disease was furthermore found in around 36% of all patients [42]. These differences in conversion rate for cancer and serious non-malignant disease between sites and nationally between the UK and Denmark, could be explained by several factors. However, the most important factor is likely the difference in threshold for referral, which was allowed by design in both the UK pilot sites and in the implementation of the NSSC-CPP in Denmark. Indeed, there still exists both inter- and intraregional differences in the implementation of the NSSC-CPP in Denmark, where patients for example, can be referred by the GP either before or after diagnostic imaging [51, 52]. As the NSSC-CPP crude referral rate and the conversion rate is only measured by the patients indeed being referred to the Diagnostic Centres and not those that are handled by the GP outside of the NSSC-CPP, such differences can lead to large differences in outcome data between local sites and between countries. In addition, since most outcome parameters are currently not monitored by the official monitoring process in the Nordic countries, only local sporadic studies such as ours are available. Therefore, the obvious next step seems to be the establishment of robust national monitoring in the Nordic countries and in other countries seeking to introduce a NSSC-CPP. This monitoring should preferably include the complete patient trajectory from the first NSSC symptoms to final diagnosis hereby enabling fine-tuning of the NSSC-CPP to secure timely and accurate diagnosis for the benefit of the patients.

With a now high and stable crude referral rate and conversion rate, the local NSSC-CPP is involved in diagnosing cancer in 6% of all cancer cases in the area of Funen. This suggests that the Diagnostic Centre can handle the highly heterogeneous patient pool presenting with various symptoms and comorbidities through the Danish NSSC-CPP. As the Diagnostic Centres in Denmark furthermore handle the diagnostic work-up of patients with suspected metastases without known primary tumour, the proportion of new cancer cases handled in total by our Diagnostic Centre is closer to 10% of all new cancer cases (unpublished data). Giving the relative low number of doctors employed at each Diagnostic Centre, the Diagnostic Centres could therefore likely be very cost-effective at handling a vital task in the diagnosis of cancer in Denmark, though this remains to be verified in subsequent health economic assessment studies.

We also showed specific blood test items that are correlated with cancer, though the individual predictive values of these are rather low. Giving the increasing number of elderly in Denmark and thus increasing number of suspected cancer referrals, further research is needed to determine if more predictive factors, including combinations of blood test items or other clinical parameters can predict cancer or other serious disease in general practice and thus help guide referral to the NSSC-CPP and limit the use of the NSSC-CPP in patients without cancer or other serious disease. These studies could employ more advanced methods such as artificial intelligence to increase the predictive value of the models.

With the inclusion of the NSSC-CPP in Denmark, an unmet medical need has been covered for patients suspected for cancer presenting with serious non-specific symptoms. However, a previous study has shown that the diagnosis of around three out of four new cancer cases in Denmark involves the patient’s GP, where around 19% of these patients present with serious non-specific symptoms that might be cancer [16]. This indicates that though we find 6% of all new cancer cases through the NSSC-CPP, this is still below half of all patients presenting with serious symptoms of cancer in general practice highlighting the need for further improvements in referral practice and outcome monitoring. In addition, patients with serious symptoms and patients with alarm symptoms only represent around two-thirds of all patients with undiagnosed cancer presenting in general practice [16]. The remaining group is found in the so-called “low but not no risk” patient group with less severe and less defined symptoms, who are probably a continuum of the NSSC-CPP population regarding symptoms and comorbidities [53], which is a large patient group that is currently ill-defined and not well studied in Denmark or beyond and thus potentially underserved.

In conclusion, this study describes the outcome of the NSSC-CPP over time from 2014 to 2021 in a local setting covering 9% of the Danish population, where we found a now consistent proportion of patients with cancer and serious non-malignant disease in an otherwise heterogeneously symptomatic population. Similar urgent referral programmes in other countries might thus fill an unmet medical need for patients presenting with serious non-specific symptoms and signs of cancer in general practice and in the secondary sector. Further work is needed to identify potential markers of cancer and serious disease and to identify differences in the utilisation and outcome of the NSSC-CPP between countries, regions, and Diagnostic Centres, but also between individual GPs in the catchment area of each Diagnostic Centre.

### Supplementary information

Supplemental material

## Data Availability

The datasets generated and analysed during this study are not publicly available due to Danish law prohibiting publishing of individual-level data but are available from the corresponding author on reasonable request following a data transfer agreement according to Danish law.
