# Supplementary material for: Risk of cancer and serious disease in Danish patients with urgent referral for serious non-specific symptoms and signs of cancer in Funen 2014–2021
Source: Br J Cancer. 2024 Feb 26;130(8):1304–15. doi: 10.1038/s41416-024-02620-y (PMC11014902; doi:10.1038/s41416-024-02620-y)
Supplement: Supplementary file 1 — Supplemental material [file 41416_2024_2620_MOESM1_ESM.docx]

**
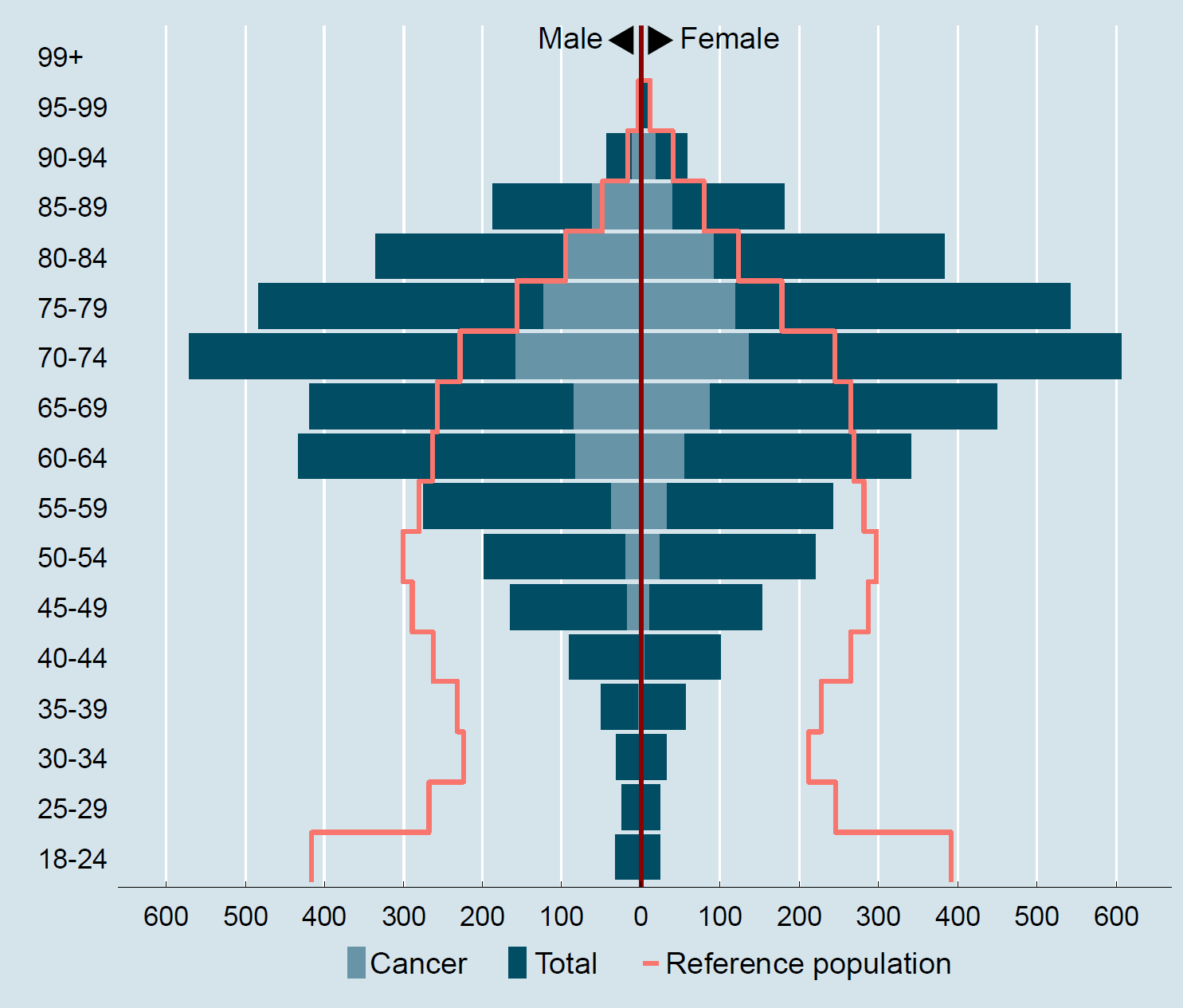
Supplemental information**

**Supplemental Figure 1:** Total population age structure of the patients referred to the NSSC-CPP of the Diagnostic Centre in Svendborg. The bars indicate the number of patients in total (dark blue) and number of patients with cancer (light blue) in each age group in total in the period 2014-2021. The orange line indicates the age distribution of the reference population of Funen (mean population numbers counted on the 1^st^ of January each year from 2014 to 2021[31]).

| **Blood test item** | **Unit** | **Abnormal values** | | | **Critical values** |
| --- | --- | --- | --- | --- | --- |
|  |  | **Male** | **Female** | **Both gender** | **Both gender** |
| ALAT | U/l | >70 | >45 |  | 2 x ref |
| ALP | U/l |  |  | >105 | 2 x ref |
| B12 | pmol/l |  |  | <200 / >600 | <150 />1000 |
| Bilirubin | µmol/l |  |  | >25 | 2 x ref |
| Ca total | mmol/l |  |  | <2.20 / >2.55 | <1.5 / >2.75 |
| Ca++ | mmol/l |  |  | <1.18 / >1.32 | <1.1 / >1.4 |
| Creatinine | µmol/l | >105 | >90 |  | N/A |
| CRP | mg/l |  |  | >10 | 2 x ref |
| eGFR | ml/min/1.73m^2^ |  |  | <60 | <30 |
| ESR | mm | >15 (18-50yo) / >20 (>50yo) | >20 (18-50yo) / >30 (>50yo) |  | 2 x ref |
| GGT | U/l | >80 (18-40yo) / >115 (>40yo) | >45 (18-40yo) / >75 (>40yo) |  | 2 x ref |
| Hb | mmol/l | <8.3 | <7.3 |  | <6.0 |
| HbA1c | mmol/mol |  |  | >47 | >60 |
| M component | Yes/no |  |  | Yes | N/A |
| LDH | U/l |  |  | >205 (<70yo) / >255 (≥70yo) | 2 x ref |

**Supplemental Table 1.** Limits used for abnormal and critical blood values. Abnormal values were defined as values outside reference intervals, while critical values were defined as very abnormal values that raise a high suspicion of serious disease including cancer. Abbreviations: Not applicable (N/A), years old (yo).

|  |  | **Blood findings** | | |  |
| --- | --- | --- | --- | --- | --- |
|  |  | **Abnormal** | **No abnormal** | **Missing data** | **Total** |
| **Imaging findings** | **Abnormal** | 965 out of 2914 (33.1%) | 80 out of 527 (15.2%) | 50 out of 184 (27.2%) | 1095 out of 3625 (30.2%) |
|  | **No abnormal** | 80 out of 1748 (4.6%) | 8 out of 547 (1.5%) | 6 out of 139 (4.3%) | 94 out of 2434 (3.9%) |
|  | **No scan** | 37 out of 224 (16.5%) | 6 out of 62 (9.7%) | 8 out of 67 (11.9%) | 51 out of 353 (14.4%) |
|  | **Not described** | 3 out of 26 (11.5%) | 0 out of 3 (0.0%) | 109 out of 257 (42.4%) | 112 out of 286 (39.2%) |
|  | **Total** | 1085 out of 4912 (22.1%) | 94 out of 1139 (8.3%) | 173 out of 647 (26.7%) | **1352 out of 6698 (20.2%)** |

**Supplemental Table 2:** Cross-tabulation of imaging and blood findings. Data is presented as number of patients with cancer out of the total number of patients in each category. Abnormal imaging findings were defined as at least one abnormal imaging finding on either CT, PET/CT, ultrasound, or X-ray performed within 12 weeks prior to first visit or during the investigational course. Patients with more than one imaging type performed were only required to have at least one abnormal finding on either of the imaging types to be categorised as abnormal. “No scan” indicates that no diagnostic imaging was performed within the specified time period. “Not described” indicates that diagnostic imaging was performed during the period, but the results of the scan has not been categorised as either abnormal or no abnormal findings. Abnormal blood findings were defined as at least one abnormal blood finding (value outside reference interval as defined in Supplemental Table 1) in any of the 15 included blood test items. “Missing data” indicates that values for less than 10 blood test items were available for analysis for the given patients.

|  | **Main cancer diagnosis** | **Stage main** | **Second primary cancer diagnosis** | **Stage 2^nd^** | **Third primary cancer diagnosis** | **Stage 3^rd^** | **Survival status at 6 months follow-up** |
| --- | --- | --- | --- | --- | --- | --- | --- |
| Patient 1 | Chronic leukaemia | Stage B ^*^ | Prostate cancer | Stage 1 ^‡^ |  |  | Alive |
| Patient 2 | Chronic leukaemia | Stage A ^*^ | Prostate cancer | Stage 3 ^‡^ |  |  | Alive |
| Patient 3 | DLBC lymphoma | Stage 4 ^†^ | Breast cancer | Stage 1 ^‡^ |  |  | Alive |
| Patient 4 | Lung cancer | Stage 2 ^‡^ | Multiple myeloma | Stage 1 ^#^ |  |  | Alive |
| Patient 5 | Ovarian cancer | Stage 1 ^‡^ | Breast cancer | Stage 1 ^‡^ |  |  | Alive |
| Patient 6 | DLBC lymphoma | Stage 1 ^†^ | Breast cancer | Stage 1 ^‡^ |  |  | Alive |
| Patient 7 | Gallbladder cancer | Stage 4 ^‡^ | Kidney cancer | Stage 1 ^‡^ |  |  | Deceased |
| Patient 8 | Pancreatic cancer | Stage 4 ^‡^ | Breast cancer | Stage 1 ^‡^ |  |  | Deceased |
| Patient 9 | Lung cancer | Stage 4 ^‡^ | Breast cancer | Stage 1 ^‡^ |  |  | Alive |
| Patient 10 | Hodgkin lymphoma | Stage 4 ^†^ | Lung cancer | Stage 1 ^‡^ |  |  | Alive |
| Patient 11 | Follicular lymphoma | Stage 1 ^†^ | Malignant melanoma | Stage 1 ^‡^ |  |  | Alive |
| Patient 12 | Small bowel cancer | Stage 1 ^‡^ | Pancreatic cancer | Stage 1 ^‡^ |  |  | Alive |
| Patient 13 | Lung cancer | Stage 4 ^‡^ | Multiple myeloma | Stage 1 ^#^ |  |  | Alive |
| Patient 14 | DLBC lymphoma | Stage 4 ^†^ | Breast cancer | Stage 1 ^‡^ |  |  | Alive |
| Patient 15 | Polycythaemia vera | N/A | B cell lymphoma | Stage 4 ^†^ |  |  | Alive |
| Patient 16 | Colon cancer | Stage 2 ^‡^ | Lung cancer | Stage 1 ^‡^ | DLBC lymphoma | Stage 1 ^†^ | Alive |
| Patient 17 | Colon cancer | Stage 2 ^‡^ | Multiple myeloma | Stage 1 ^#^ |  |  | Alive |
| Patient 18 | Gallbladder cancer | Stage 4 ^‡^ | Breast cancer | Stage 2 ^‡^ |  |  | Alive |
| Patient 19 | Gallbladder cancer | Stage 4 ^‡^ | Multiple myeloma | Stage 1 ^#^ |  |  | Deceased |
| Patient 20 | Lung cancer | Stage 4 ^‡^ | Oesophageal cancer | Stage 1 ^‡^ |  |  | Alive |
| Patient 21 | Lung cancer | Stage 3 ^‡^ | HCC | Stage 1 ^‡^ |  |  | Alive |
| Patient 22 | DLBC lymphoma | Stage 1 ^†^ | Colon cancer | Stage 3 ^‡^ |  |  | Alive |

**Supplemental Table 3:** Overview of the 22 patients that had one or more second primary cancers during the 6 months of follow-up. The main cancer diagnosis was selected based on severity of the cancer and associated treatment and/or course of disease (including cause of death). ^*^ Chronic lymphocytic leukaemias (CLL) were staged according to the Binet staging system. ^†^ Lymphomas were staged according to the Ann Arbor staging system. ^‡^ Solid tumours were staged according to the 8^th^ edition of the UICC TNM staging system. ^#^ Multiple myelomas were staged according to the Revised International Staging System (RISS). Abbreviations: diffuse large B cell lymphoma (DLBC lymphoma), hepatocellular carcinoma (HCC), not applicable (N/A).
